# The Application of Metabolomics in Recent Colorectal Cancer Studies: A State-of-the-Art Review

**DOI:** 10.3390/cancers14030725

**Published:** 2022-01-30

**Authors:** Andrew Gold, Fouad Choueiry, Ning Jin, Xiaokui Mo, Jiangjiang Zhu

**Affiliations:** 1 Human Nutrition Program, James Comprehensive Cancer Center, The Ohio State University, Columbus, OH 43210, USA; gold.179@osu.edu (A.G.); choueiry.2@osu.edu (F.C.); 2 Department of Medical Oncology, James Comprehensive Cancer Center, The Ohio State University, Columbus, OH 43210, USA; Ning.Jin@osumc.edu; 3 Department of Biomedical Informatics, Center for Biostatistics, The Ohio State University, Columbus, OH 43210, USA; Xiaokui.Mo@osumc.edu

**Keywords:** colorectal cancers, metabolite biomarker, metabolomics, LC-MS, GC-MS

## Abstract

**Simple Summary:**

Colorectal Cancer (CRC) is one of the leading causes of cancer-related death in the United States. Current diagnosis techniques are either highly invasive or lack sensitivity, suggesting the need for alternative techniques for biomarker detection. Metabolomics represents one such technique with great promise in identifying CRC biomarkers with high sensitivity and specificity, but thus far is rarely employed in a clinical setting. In order to provide a framework for future clinical usage, we characterized dysregulated metabolites across recent literature, identifying metabolites dysregulated across a variety of biospecimens. We additionally put special focus on the interplay of the gut microbiome and perturbed metabolites in CRC. We were able to identify many metabolites showing consistent dysregulation in CRC, demonstrating the value of metabolomics as a promising diagnostic technique.

**Abstract:**

Colorectal cancer (CRC) is a highly prevalent disease with poor prognostic outcomes if not diagnosed in early stages. Current diagnosis techniques are either highly invasive or lack sufficient sensitivity. Thus, identifying diagnostic biomarkers of CRC with high sensitivity and specificity is desirable. Metabolomics represents an analytical profiling technique with great promise in identifying such biomarkers and typically represents a close tie with the phenotype of a specific disease. We thus conducted a systematic review of studies reported from January 2012 to July 2021 relating to the detection of CRC biomarkers through metabolomics to provide a collection of knowledge for future diagnostic development. We identified thirty-seven metabolomics studies characterizing CRC, many of which provided metabolites/metabolic profile-based diagnostic models with high sensitivity and specificity. These studies demonstrated that a great number of metabolites can be differentially regulated in CRC patients compared to healthy controls, adenomatous polyps, or across stages of CRC. Among these metabolite biomarkers, especially dysregulated were certain amino acids, fatty acids, and lysophosphatidylcholines. Additionally, we discussed the contribution of the gut bacterial population to pathogenesis of CRC through their modulation to fecal metabolite pools and summarized the established links in the literature between certain microbial genera and altered metabolite levels in CRC patients. Taken together, we conclude that metabolomics presents itself as a promising and effective method of CRC biomarker detection.

## 1. Introduction

Colorectal Cancer (CRC) is the second leading cause of cancer-related death when male and female data are combined [1]. According to the American Cancer Society, colon and rectal cancers combined are projected to amount to 149,500 new cases and 52,980 related deaths in 2021 [1]. Individual colorectal tumors are typically present as adenocarcinomas originating from the epithelial cells of the colonic mucosa [2]. CRC can stem from mutations in a wide variety of genes, including *adenomatous polyposis coli* (APC), Kirsten *rat sarcoma viral oncogene homolog* (KRAS), *tumor protein 53* (TP53), and genes relating to chromosomal instability and DNA mismatch repair [3]. These genes can lead to significant dysregulation of genetic and metabolic processes, including dysregulated amino acid metabolism driven by APC mutations and dysregulation in glycolytic and glutamine pathways spurred by mutations in KRAS [4,5]. Overall incidence of CRC is increasing in Americans under 50 (early onset CRC), but death rates are dropping due to improved screening techniques [1,6]. The major techniques employed in CRC screening and detection include colonoscopy, tissue biopsy, and fecal occult blood test (FOBT) [7]. These techniques are invasive and can be uncomfortable for the patient in the cases of colonoscopy or biopsy, or typically exhibit low sensitivity in the case of the FOBT [8]. This demonstrates the clinical need for a less invasive test with increased sensitivity.

Meanwhile, high throughput “omics” techniques such as metagenomics, transcriptomics, proteomics, and metabolomics, offer a potentially less invasive alternative for CRC diagnosis. Each of these techniques offers its own advantages to cancer biomarker discovery and diagnosis. Genomics, for example, is particularly effective for determining CRC susceptibility and familial risk for the disease, but holds little diagnostic power as DNA sequences rarely directly translate to phenotype due to epigenetic, post-transcriptional and post-translational modifications [9]. Transcriptomics and proteomics represent closer ties to the phenotypic state of the organism, and, especially when integrated, hold some diagnostic power [10]. However, their individual diagnostic power falls short of that of metabolomics, which allows for time-sensitive and accurate phenotypic profiling of the organism and its metabolic pathways, as well as the ability to analyze the interplay between host & gut bacterial metabolites, which represents an integral part of CRC pathogenesis [10]. Metabolomics is defined as “the comprehensive analysis of metabolites (small molecule intermediates or end products of metabolic processes) in a biological specimen [6,11].” Metabolomics is typically conducted with either NMR spectroscopy or a form of coupled chromatography–mass spectrometry, allowing for comprehensive analysis of an individual’s metabolic phenotype [6]. Researchers have employed metabolomics techniques to identify biomarkers of a multitude of diseases with high specificity and sensitivity, including several types of cancer [12,13,14]. Metabolomics can be conducted over a wide range of biological specimens, many of which can be collected with minimally invasive techniques. However, biomarker specificity and metabolite identification often can fluctuate based upon specimen, stage of colorectal cancer, and even between different studies [15]. In accordance, many primary studies have been conducted attempting to identify commonalities in the metabolic profiles of CRC patients using metabolomics analyses.

A small number of review papers drawing conclusions from multiple metabolomics studies in CRC have been published over the years. Erben et al. characterized a wide breadth of primary studies, especially well-characterizing differences in metabolite profiles between biological specimen types [12]. Zhang et al. reviewed a smaller breadth of literature but provided substantial pathway analysis relating to potential markers of CRC incidence [16]. Yusof et al. characterized studies addressing metabolic profile differences specifically between stages of CRC [17]. Hashim et al. additionally reviewed serum specimen-only metabolomics studies, providing a collection of specific data applicable to this particular specimen [18]. While each of these reviews has contributed greatly to the understanding of metabolomics analysis of CRC patients, many lack a clear and easy-to-read condensation of metabolites identified across multiple primary studies. Additionally, the contribution of the gut microbial population and microbial metabolites to CRC pathogenesis has been identified as crucial in many recent studies [19,20]. This important connection, however, has largely been overlooked by reviews of metabolomics profiling in CRC. Accordingly, we attempted to provide a comprehensive analysis of current literature in the field of metabolomics identification of CRC biomarkers, comparing metabolomics biomarkers of CRC across different biospecimens, while paying close attention to fluctuations in gut microbial composition and their associated changes in the metabolome in stool specimen studies. We expect our review will provide clear analysis of recent studies in the field, offering easy-to-read tabulation of commonly identified metabolites differentially regulated in CRC patients, as well as characterizing microbial contributions to the CRC metabolome, which collectively should present a strong foundation for further research in metabolomics profiling of CRC.

## 2. Methods

### 2.1. Systematic Literature Review 

**Figure 1:**
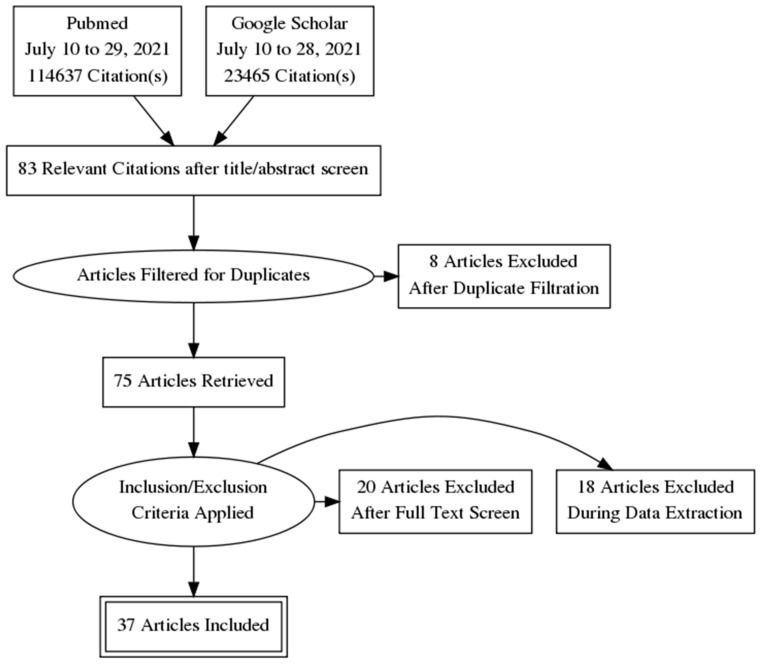
PRISMA flow diagram detailing the literature review process. After filtration, 37 relevant articles were included.

We conducted multiple sets of systematic literature search using both Google Scholar and PubMed databases from 10 to 29 July 2021. We first used the terms: (biomarker OR biomarkers OR metabolite OR metabolites OR metabolome OR metabolomic OR metabolomics OR metabolic) AND (“Colorectal neoplasm” OR “colon neoplasm” OR “colonic neoplasm” OR “Rectal Neoplasm” OR “colorectal cancer” OR “colon cancer” OR “colonic cancer” OR CRC OR “Colorectal tumor” OR “colon tumor” OR “colonic tumor” OR adenoma)), then (biomarker OR biomarkers OR metabolite OR metabolites OR metabolome OR metabolomic OR metabolomics OR metabolic) AND (“early onset” OR “sporadic” OR “late onset”) AND (“Colorectal neoplasm” OR “colon neoplasm” OR “colonic neoplasm” OR “Rectal Neoplasm” OR “colorectal cancer” OR “colon cancer” OR “colonic cancer” OR CRC OR “Colorectal tumor” OR “colon tumor” OR “colonic tumor” OR adenoma)), then (biomarker OR biomarkers OR metabolite OR metabolites OR metabolome OR metabolomic OR metabolomics OR metabolic) AND (“polyp” OR “colorectal polyp” OR “Adenomatous polyp” or “colon growth”) AND (“Colorectal neoplasm” OR “colon neoplasm” OR “colonic neoplasm” OR “Rectal Neoplasm” OR “colorectal cancer” OR “colon cancer” OR “colonic cancer” OR CRC OR “Colorectal tumor” OR “colon tumor” OR “colonic tumor” OR adenoma)) and filtered results. The Preferred Reporting Items for Systematic Reviews and Meta-Analyses (PRISMA) statement flow diagram for systematic reviews was used to depict number of sources at each phase and rationale for exclusion (Figure 1) [21]. Cross references identified from original papers and reviews were also included.

Basic statistical analyses among groups of studies were conducted with Student’s two-sample t-test, assuming unequal variance, or one-way ANOVA analysis. These analyses were performed to compare number of total dysregulated metabolites identified in groups of varying statistical cutoff methods, instrument methods, specimen types, and the number of participants to identify if any of these features were relevant factors in the identification of metabolite biomarkers. 

### 2.2. Exclusion Criteria

We removed study duplicates and articles unavailable in English, and then screened remaining articles for eligible studies according to our criteria. Exclusion criteria included topics unrelated to the review question, review articles, studies unavailable for open-access reading, and studies that were not focused on metabolomics-based biomarker detection in human subjects. Studies were included if published from January 2012 to July 2021.

## 3. Results & Discussion

### 3.1. Overview

**Figure 2:**
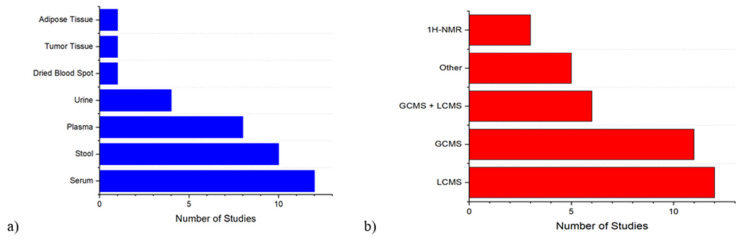
(**a**) Breakdown of the number of studies by specimen type. (**b**) Breakdown of the number of studies by platform of analysis.

In this study, relevant articles after PRISMA filtration were reviewed and patient data was retrieved and classified to provide context for further study breakdown. The biological specimen, stage of CRC, number of cases of CRC and controls, analysis platform, patients age, stage, country of origin, and year of study were recorded (Table 1, Figure 2). 

**Figure 3:**
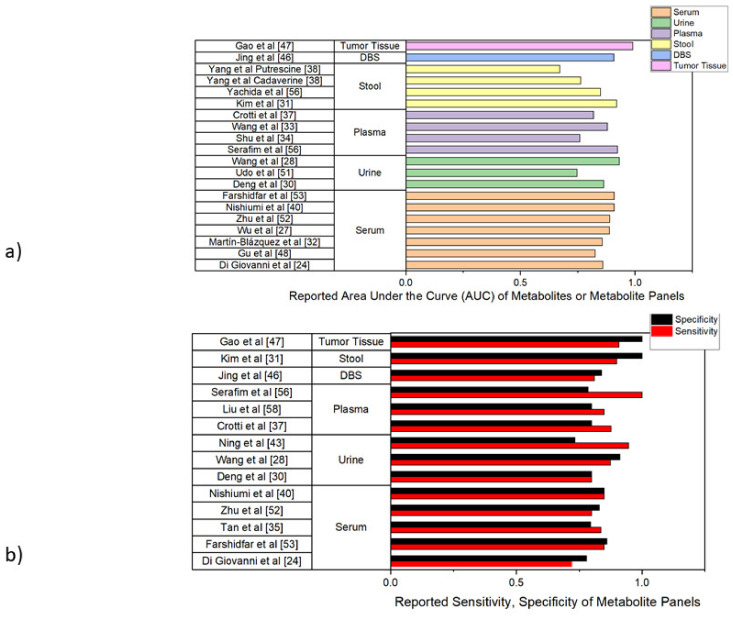
(**a**) Summary of the areas under the receiver operating curve (AUCs) for CRC diagnostic models created using identified metabolite biomarkers in each reference. References are stratified by specimen type. (**b**) Sensitivity and Specificity of diagnostic models created using metabolite biomarkers when listed. References are stratified by specimen type. DBS = Dried Blood Spot.

Studies were then compared for differentially detected metabolites between CRC and control populations under reported statistical threshold of each study and metabolites were classified according to several major molecular classes (Table 2), while any study reporting testing of a diagnostic model was evaluated by area under the receiver operating curve (AUC), sensitivity, and specificity. These are summarized in Figure 3.

Metabolites of particular interest (identified in three or more studies) were reviewed to see whether they were most commonly reported as increased or decreased in CRC compared to control (Table 3).

**Figure 4:**
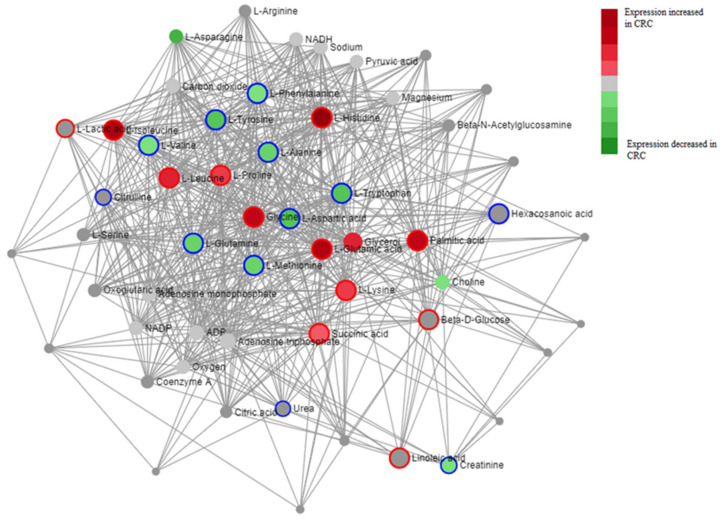
Diagram depicting a metabolite–metabolite interaction network for major metabolites identified to be differentially regulated across studies. Map was created using MetaboAnalyst’s network analysis feature, using a degree filter of 5.0 and betweenness cutoff of 2.0. Color of metabolite represents directionality of difference between CRC and control in literature (Difference was calculated by number of papers identifying metabolite as upregulated—number of papers identifying metabolite as downregulated). Nodes are connected utilizing the KEGG database of metabolic pathways, with larger nodes being implicated in more pathways and thus having more connections. Outline of metabolite represents pathways that are up or downregulated in CRC, analyzed using the KEGG database, with blue-outlined metabolites representing pathways downregulated in CRC and red-outlined metabolites representing pathways upregulated in CRC.

Additionally, differentially regulated metabolites in CRC were then mapped in accordance with their implicated metabolic pathways in order to visualize metabolic networks perturbed in CRC pathogenesis (Figure 4, Table 4).

**Figure 5:**
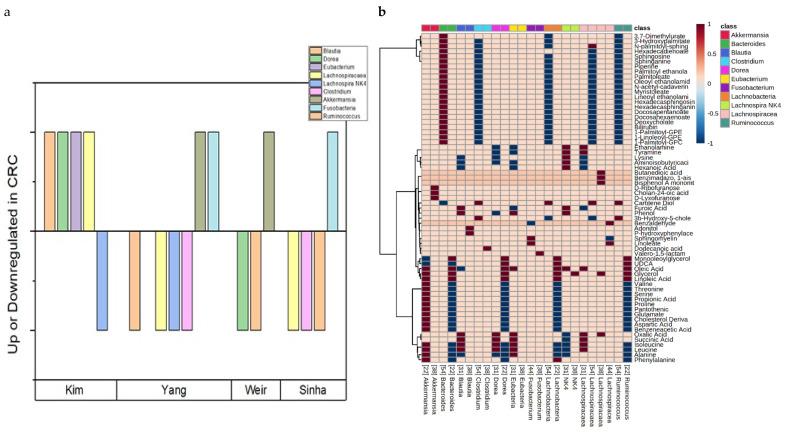
(**a**) Graphical representation of bacterial genera identified to be differentially regulated in multiple studies over four studies reporting that data [33,37,44,48]. A bar above the x axis indicates upregulation of that bacterial genus in CRC fecal tissue, while a bar below the x axis indicates downregulation. (**b**) Heatmap demonstrating the identified Pearson correlation of bacterial genus identified as differentially regulated in multiple sources and identified metabolites in five studies [33,34,37,44,48], using stool as the primary specimen. A positive (>0) value on the heatmap implies a positive correlation between bacterial genus and metabolite, while a negative (<0) value implies negative correlation between the genus and that metabolite. A 0 indicates no reported correlation for that metabolite.

Lastly, six studies focusing on the metabolome from stool specimens and their corresponding microbial populations were further analyzed to shed light on the gut bacterial population’s impact on the metabolic profile of CRC (Figure 5).

### 3.2. Study Design and Population Characteristics

After two of the authors independently evaluated the literature following our workflow in Figure 1, we identified a total of 37 relevant articles after applying the reported inclusion and exclusion criteria. Population characteristics, methods of analysis (platform), the year of study, and country of origin of these selected studies are reported in Table 1. Studies with incomplete or missing information for a particular category are marked N/A (Table 1). These studies ranged through a variety of populations, including CRC patients from USA, China, Japan, Italy, Canada, Belgium, Singapore, Germany, Romania, South Korea, France, and Brazil, although most studies were clustered in North America or East Asia (Table 1). Samples were extracted from a variety of biological sources, including serum (12 studies), stool (10 studies), plasma (8 studies), urine (4 studies), tumor tissue (1 study), adipose tissue (1 study), and dried blood spot (1 study) (Figure 2a). Populations of these studies were generally small, but had a large spread, ranging from 10 CRC cases with matched control [44] to 744 total patients [22]. Five studies had less than 50 total participants, eight studies had 50–100 participants, eight had 100–200 participants, and sixteen had more than 200 participants. Patient counts were broken down by training and validation sets when available (Table 1). Ages of study participants ranged from 18–92 in studies where age ranges were available (Table 1). Here we describe age data in the most detailed form it was reported in by the study in question, either by age range or mean/median age, either overall or within specified study groups such as control or CRC. Additionally, some studies reported age ranges or mean/median ages by stages of CRC. We reported gender as percentage of total subjects that were male, which was available in nearly all studies (Table 1). Gender percentages for the entire study were calculated for studies only reporting gender data by stage of CRC, broken down by control or CRC groups, or separated by training and validation sets. Studies ranged from 24% male [27] to 77% male [57], although most studies ranged between 45% and 65% male participants.

It is interesting to note that almost all studies identified in our review represented clinical studies with diagnostic aims (including detailed staging attempts), highlighting the need for better diagnostic approaches in CRC, while one study monitored the treatment/remission process of CRC [27]. Most studies employed a case-control research design, using either a healthy (carcinoma-free) control, adenomatous polyp group, or both. Twenty-one studies compared CRC directly to healthy control [24,25,26,31,33,36,38,39,42,43,44,45,46,48,49,50,51,52,53,54,56]. Jing et al. and Gao et al., conversely, compared CRC patients to an adenomatous polyp control [32,55] while eleven studies utilized CRC, healthy control, and adenomatous polyp groups [28,29,30,34,35,37,40,41,47,57,58]. Two studies (Geijson et al., Liu et al.) did not include a control group [22,23], while Di Giovanni et al. compared CRC groups to healthy control in both CRC models as well as CRC remission models, using separate controls but comparing CRC-remission and CRC patients using an Effect Size model [27]. This allowed the group to differentiate metabolite profiles between pre- and post-treatment CRC patients. Of note, special attention was also paid to distinguish early and late onset CRC [42], to stratify biomarkers by CRC stage [22,23,28], and to correlate genetic analysis of the gut microbiota with metabolite alterations in CRC [33,34,37,44,46,48]. For example, Holowatyj et al. [31] compared early and late-onset CRC to healthy control and identified 13 dysregulated metabolites in young-onset patients and 103 dysregulated metabolites in late-onset patients [31], while 35 metabolites were detected differentially in early vs. late-onset CRC patients [26]. For the purposes of our study, these datasets were combined to one large CRC group, but it is worth noting that early and late-onset CRC metabolic profiles significantly differ, with further metabolic dysregulation occurring in late-onset patients [31]. Meanwhile, both Geijson et al. and Liu et al. opted for pairwise comparison of metabolomic profiles in different stages of CRC highlighting several metabolites that were differentially regulated in specific stages of CRC [22,23]. These included citrulline, histidine, and several lysophosphatidylcholine molecules, all of which were found in lower concentrations in later stage CRC, as well as several triglycerides which were found in higher concentrations in later stage CRC [22,23]. Also, Farshidfar et al. distinguished metabolites between different CRC stages and healthy control, but did not compare metabolites among stages of CRC [28]. It is important to note that these studies show the usefulness of metabolomics beyond just CRC diagnosis, as it can be potentially used to differentiate not only healthy controls from CRC patients, but also to identify different stages of disease as well as to predict early or late onset disease. 

### 3.3. Analytical Methods and Their General Performance

In our review, most studies employed a type of mass spectrometry analysis, with three studies employing ^1^H-NMR analysis [29,42,53]. Liquid-Chromatography-Mass Spectrometry (LCMS), including Ultra-High Performance Liquid Chromatography-Mass Spectrometry (UHPLC-MS) and Liquid Chromatography-High Resolution Mass Spectrometry (LC-HRMS), was the most popular method for analysis, with eighteen identified studies that applied this technique [22,23,24,26,30,31,33,35,36,38,39,41,43,47,48,49,50,52], followed by Gas Chromatography-Mass Spectrometry, which was conducted in seventeen studies [24,25,27,28,34,38,39,44,45,46,48,49,50,51,54,56,57] (Figure 2b). These counts include four studies conducting both LCMS and GCMS. Other methods of analysis included direct infusion mass spectrometry (1 study) [32], Capillary electrophoresis-time of flight mass spectrometry (CE-TOFMS) (3 studies) [37,40,55], and Matrix Assisted Laser Desorption Ionization-Time of Flight mass spectrometry (MALDI-TOF MS) (1 study) [58] (Figure 2b). Thirty studies [23,24,25,27,28,29,30,31,32,34,35,36,37,38,39,40,42,43,44,45,46,48,49,50,53,54,56,57,58] employed untargeted metabolomic profiling, while 9 studies employed targeted metabolomics, either with a metabolite subset in mind to begin the study or as part of a validation of their untargeted data [22,25,26,28,35,47,51,52,55]. Of studies where the selections of analytical columns could be identified, 16 used a column that could identify polar and nonpolar compounds [22,25,26,27,28,31,33,36,38,39,44,48,49,51,55,58], 8 focused on polar compounds [23,24,30,34,47,52,54], and 2 used nonpolar compounds [45,46]. Additionally, six studies [23,25,52,56,57,58] conducted lipidomics, a subset of metabolomics specifically targeted at lipid identification, as the lipidome is often perturbed in CRC patients [59]. Song et al., in addition to finding two individual fatty acids expressed differently in CRC compared to control in male participants, also found that the total levels of ω-6 polyunsaturated fatty acids as well as total monounsaturated fatty acids were raised in male CRC patients compared to control [57]. Total number of metabolites identified as biomarkers after filtering with statistical cutoff ranged from one (Cross et al.) to 74 (Holowatyj et al.) [24,31]. Total number of metabolites identified after statistical screening did not significantly differ from that of one-way ANOVA analysis using LCMS, GCMS, ^1^H-NMR, or other instrument methods. Total metabolite counts for each study are listed in Table 2.

### 3.4. Evaluating the Performance of Metabolomics-Based Assays in CRC Studies

To understand the overall performance of metabolomics assays for CRC diagnosis and differentiations, we evaluated the sensitivities, specificities, and AUC values based on available data provided in the included studies. Metabolites associated with CRC risk for each study at some levels of statistical significance and their general compound class are listed in Table 2. In these studies, comparisons were made in terms of metabolite regulation between: (i) a CRC group and a healthy control group, (ii) a CRC group and an adenomatous polyp group, or (iii) two different stages of CRC patient, where the latest available stage of CRC was compared to the earliest available stage of CRC. Metabolites differentially regulated under the statistical cutoff reported by the study in question were counted, sorted for direction of regulation (i.e., whether they were up or downregulated in CRC), and tabulated based on compound class in Table 2. Statistical threshold using *p*-value, Bonferroni corrected *p*-value or false discovery rate (FDR) for metabolite inclusion reported by each study is listed in the final column of Table 2. Six studies used a different *p*-value to represent statistical significance (0.1, 0.01, 0.001, or 0.005) [27,33,37,40,51,55]. Reasonings for increased or decreased *p*-value threshold differed. For example, Di Giovanni et al. employed several different *p*-values, including 0.01, 0.05, and 0.1, but considered any value below 0.1 statistically significant [27]. Yachida et al. employed a Mann–Whitney *U*-test for statistical analysis and thus also used a *p*-value of 0.005 cutoff [37]. Gao et al. used a targeted approach to characterize mainly amino acids’ contribution to the CRC metabolome and employed a *p* value < 0.001, as this statistical cutoff encompassed metabolites that showed the highest individual sensitivity and specificity in their validation set [55]. Kim et al. and Nishiumi et al. additionally employed an FDR-adjusted *p*-value of 0.1, while Uchiyama et al. employed a *p*-value of 0.01 [34,40,51]. None of these three authors offered extensive in-text justification for these values. Some studies also employed a Partial Least Squares Discriminant Analysis (PLS-DA) VIP score cutoff of >1 for metabolite inclusion of diagnostic models [29,36,39,42,43,47,58]. There was no significant difference in total metabolites identified between simple *p*-value cutoff and *p*-value correction or VIP score cutoff when compared with a standard two-sample *t*-test (*p* = 0.74 vs. adjusted *p* value, *p* = 0.51 vs. VIP cutoff). As summarized in Table 2, number of metabolites reported to be differentially regulated in the CRC population varied widely among different studies. For instance, Cross et al. were only able to identify one metabolite (leucyl-leucine) associated with CRC risk in both men and women, but significance was not below a Bonferroni-corrected *p* value of 0.05 [22], while Holowatyj et al. identified 116 dysregulated metabolites [31]. From these diverse studies, it seems that the wide range in the number of metabolites meeting statistical threshold, in addition to not being due to statistical cutoff value, could also not be attributed entirely to instrument type (LCMS, GCMS, ^1^H-NMR, Other) or specimen type (plasma, serum, stool, urine, dried blood spot) when analyzed using one-way ANOVA analysis (*p* values of 0.889, 0.509 for the separate means model of instrument type and specimen type respectively). There was a general trend of identification of larger metabolite sets with increasing sample size when compared with two sample t-test, but differences were slightly above a *p* value of 0.05. For instance, studies including less than 40 CRC patients identified fewer metabolites than studies with 200+ CRC patients, but only at *p* = 0.13. When studies were stratified into two larger groups, those with 0–60 CRC patients and those with 61+ patients (split so there would be roughly an equal number of studies in each group), studies with 61+ patients identified an average of 10 more metabolites, at *p* = 0.11. Variation of metabolites, in addition to sample size, together with differences in instrument, technique, and study population at different geographic locations (i.e., patients in Japan may have different metabolic profiles than those in America) might explain why different metabolites were identified by various studies [60].

### 3.5. Diagnostic Model Performance 

Some studies were able to build diagnostic models capable of differentiating between CRC patients and healthy controls with a small number of individual metabolites, while the majority identified larger panels of dysregulated metabolites. Overall, these studies identified between 1 and 116 total dysregulated metabolites (Table 2).

These metabolites encompassed a wide range of compound classes which differed by specimen, technique, sample size, and aim of the study. Only a select number of studies constructed statistical diagnostic models using these dysregulated metabolites. For instance, Yang et al. identified several metabolites as differentially regulated in CRC but only employed two, lysine breakdown products putrescine and cadaverine, as separate diagnostic markers for AUC analysis, claiming that polyamines such as these are widely dysregulated in CRC (Figure 3a) [46]. Interestingly, despite their insistence that the models generated by these metabolites were predictive of CRC diagnosis, no other studies identified putrescine as a dysregulated metabolite in CRC, and only one other study identified a derivative of cadaverine (n-acetyl-cadaverine) [33]. Generated AUC values were 0.672 for putrescine and 0.764 for cadaverine. ROC in other studies far better in terms of AUC value. Kim et al. used a combination of two metabolites, leucine and oxalic acid, to generate their receiver operating curve (ROC) as these metabolites gave the model the best sensitivity and specificity in their validation set [33]. Udo et al. employed a three metabolite panel using butyrate, leucine, and carnosine to generate a ROC with an AUC of 0.748 for CRC diagnostics against healthy controls (Figure 3a) [41]. Wang et al., conversely, used a panel of eight metabolites to generate their diagnostic model for differentiation of CRC patients and healthy controls [43]. Other studies employed larger panels, such as a 24-metabolite panel employed by Di Giovanni et al. to generate their ROC. The highest reported AUC values were derived by Gao et al., which derived an impressive AUC value of 0.991 using methionine, tyrosine, valine, and isoleucine and was able to distinguish between CRC and adenomatous polyp tissue using these metabolites; Kim et al., which was able to establish an AUC of 0.92 in its validation model using just leucine and oxalic acid, as well as an AUC of 1.0 combining these metabolites with their metagenomics data; Wang et al., which established an AUC of 0.933 from a panel of four metabolic biomarkers of different classes, and Serafim et al., which established an AUC of 0.924 from a two lipid metabolites (Figure 3a) [33,42,55,58]. Interestingly, there was no overlap between any metabolites driving these ROC’s. Wang et al.’s diagnostic model, in addition to reporting an AUC of 0.880 for its validation set, also tested patients’ survival using its eight diagnostic metabolites with a LASSO-risk scale [43]. Briefly, they dichotomized their 73 CRC patients into two roughly equal groups considered lower and higher risk, which were then followed for survival status [43]. The model was able to accurately predict overall survival time at a *p* value of 0.022 as well as progression-free survival time at a *p* value of 0.002 [43]. The sensitivity, specificity, or AUC are comparable between studies of different specimen types observed. Individual study AUC as well as corresponding sensitivity and specificity for diagnostic models used for biomarker validation were depicted in Figure 3a. Among these studies, Kim et al. and Gao et al. reported the highest specificity (1.0), while Serafim et al. reported the highest sensitivity (1.0) (Figure 3b) [33,55,58]. Sensitivity values ranged from 0.72 to 1.0, while specificity values ranged from 0.733 to 1.0 [27,33,54,55,58]. Average sensitivity and specificity of diagnostic models reporting these values were 0.855 and 0.839, respectively.

Generally, models employing a combination of multiple classes of metabolites, such as a combination of amino acid and lipid biomarkers, were demonstrated to hold more diagnostic power. For instance, Jing et al.’s model combining a combination of eight acylcarnitine and amino acid biomarkers had an AUC of 0.909, while Yang et al.’s single-metabolite cadaverine and putrescine models only reported AUC values of 0.765 and 0.672 respectively [32,46]. This was not an absolute rule, and while larger metabolite panels typically performed better in diagnostic validation, some smaller models such as Kim et al.’s model using only leucine and oxalic acid (0.92) [33]. The generally high sensitivity markers of the identified studies (the average sensitivity across identified studies was roughly 86% as depicted in Figure 3b) far exceed the measured sensitivity of the current diagnosis methods guaiac FOBT (sensitivity of ~65%) and immunochemical FOBT (~75%) [8]. Thus, diagnostic models created using metabolic biomarkers may be powerful predictors for CRC diagnosis. 

### 3.6. Frequently Reported Metabolite Biomarkers in CRC Studies

CRC is a disease with a complicated pathology and metabolite biomarkers may vary based upon biospecimen, stage of cancer, and method of analysis. In totality, however, there were still many metabolites commonly identified across different methods of analysis and specimens. After identification of metabolites reported as potential biomarkers of CRC development, we stratified metabolites by the number of times being identified as a statistically significant indicator of CRC risk as well as directionality of this risk (higher amounts of metabolite in CRC patients are listed as upregulated, while higher amounts of metabolite in healthy control are listed as downregulated). We reported metabolites identified in 3 or more studies as significantly differentially regulated in Table 3. For example, amino acid metabolism has been characterized as significantly altered in cancer pathogenesis, and accordingly the majority of frequently identified, differentially regulated metabolites in the identified studies were amino acids [61]. Alanine, tyrosine, asparagine, aspartic acid, tryptophan, methionine, and glutamine showed the most significant decrease in CRC patients among metabolites identified in multiple studies, while glutamic acid, glycine, histidine, and isoleucine showed significant upregulation (Table 3). Among other types of metabolites, lipids and lipid-related molecules were the most often identified groups. Palmitic acid and linoleic acid were identified in five papers each, with palmitic acid shown to be consistently upregulated across all five studies and linoleic acid upregulated in three studies as well, while linoleic acid was found to be downregulated in two studies (Table 3). Palmitic acid, a saturated fatty acid, has been more consistently associated with CRC pathogenesis in scientific literature than linoleic acid, an unsaturated fatty acid, which shows mixed results in terms of CRC correlation [62,63,64]. 3-hydroxybutyrate, a ketone body and product of fatty acid degradation, was also upregulated in CRC (Table 3), but also shows an unclear relationship with CRC, and it was indicated to drive cancer proliferation in some studies while to be better prognostic outcomes when upregulated in other studies [65,66]. Lysophosphatidylcholines of varying lengths were downregulated in CRC patients, while free choline was also found to be downregulated (Table 3). Glycerol, a byproduct of glycolysis as well as a precursor to triglyceride formation, was identified as upregulated in four studies (Table 3). Lastly, urea cycle-related metabolites such as urea, citrulline, and hippuric acid, glycolytic intermediates such as glucose and lactate, as well as TCA cycle metabolite succinate, were found to be perturbed in CRC, although there was a lack of consistency in directionality of regulation (Table 3). Many studies gave contradictory results, in which a certain metabolite may be found upregulated in CRC patients over healthy control in one study, while downregulated in another. Generally (but not always) this differential regulation could be attributed to differences in specimen. For instance, the metabolites citrulline and alanine are identified as upregulated in CRC patients in 2 and 4 studies respectively, but also identified as downregulated in 3 and 6 studies respectively (Table 3). The studies in which citrulline was found to be upregulated occurred in urine and stool, but all studies identifying downregulation occurred in blood-related specimens such as plasma, serum, and dried blood spot (Table 3). This differential may be explained by the increase in amino acid mobilization seen across studies. Citrulline is a urea cycle metabolite and would likely be excreted in urine more often during increased protein breakdown, when the urea cycle is employed for nitrogen disposal [67,68]. Conversely, this would result in lower levels of circulating citrulline in the blood. Valine, alanine, and succinate were also found upregulated in stool but down in most blood-related specimens (Table 3). The exact mechanisms for this differential remain unknown but may be related to the bacterial populations in the gut that are overproducing these metabolites being correlated to unrelated mechanisms of CRC proliferation, while endogenously produced levels of these amino acids are negatively correlated with CRC. Certain metabolites were also only identified in particular specimen types. For instance, 3-hydroxybutyrate, aspartic acid, glucose, glycerol, glycine, isoleucine, leucine, linoleic acid, lysine, serine, and sphinganine were all only discovered in serum and stool specimen studies (Table 3). Histidine was only identified in blood-related specimen (serum and plasma), while kynurenine was mostly identified in urine or stool (Table 3). Altogether, our data demonstrate that specimen type likely plays an influence in metabolite identification.

We additionally were able to show correlated metabolites through pathway interconnectivity (Figure 4, Table 4). Metabolites identified in three or more studies (Table 3) were analyzed for consensus direction of regulation in CRC vs. healthy control tissues and mapped in accordance with their level of connection to one another through related metabolic pathways (Figure 4). Metabolic pathways most frequently upregulated in CRC pathogenesis include aminoacyl-tRNA biosynthesis, valine, leucine, and isoleucine biosynthesis, and butanoate metabolism. Pathways most often downregulated also include aminoacyl-tRNA synthesis, as well as arginine biosynthesis and alanine, aspartate, and glutamate metabolism (Table 4). Notably, aminoacyl-tRNA biosynthesis was the most perturbed pathway connecting both up and downregulated metabolites in CRC patients vs. healthy controls. This likely stems from cancer’s demand for differential rates of synthesis of different proteins than normal tissues (i.e., increased need for proteins related to proliferation and cell migration and lower need for more cell-specific, specialized proteins), which would ultimately lead to some metabolites in this pathway being upregulated and some downregulated [69]. Ultimately, identified pathways were largely driven by dysregulation of amino acid metabolism, which was perturbed in many identified studies. Further study of these dysregulated pathways may begin to elucidate more detailed metabolic mechanisms for CRC pathogenesis. 

### 3.7. Metabolite Classes of Interest

The most frequently identified metabolite class perturbed in CRC tissue across all 37 identified studies was amino acids. Several major proteinogenic amino acids, including alanine, tyrosine, asparagine, aspartic acid, valine, glutamic acid, glycine, histidine, and isoleucine, were identified to be dysregulated in CRC patients across the majority of studies we identified (Table 3). While the major energy pathway perturbed in cancer metabolism is glycolysis, in what is known as the Warburg effect [70], amino acid metabolism is often also significantly altered [71,72]. Amino acids can be preferentially catabolized to feed the upregulated metabolism of cancer cells, serve as precursors for the excessive nucleotide synthesis of cancer cells, be broken down to synthesize glutathione to neutralize the increased reactive oxygen species (ROS) proliferated by cancerous cells, or used as transcriptional or epigenetic regulators to fuel cancer-specific processes [71]. For example, glycine, found to be increased in CRC samples in the majority of our identified studies (Table 3), can serve as a carbon and nitrogen donor for purine biosynthesis as well as a source of carbon for the methionine-folate cycle [71]. Many other amino acids can serve as anaplerotic substrates for continued glycolysis or TCA cycle metabolism [73]. Of additional interest in CRC is downstream tryptophan metabolite kynurenine, which can act as a ligand and induce immunosuppression via the aryl hydrocarbon receptor (AHR) [71,74]. This binding impairs the ability of dendritic cells and regulatory T cells to eliminate cancer cells [71]. In CRC, this enables increased cancer cell growth and proliferation, and inhibition of kynurenine production has been experimentally proven to limit cancer proliferation [74]. Kynurenine was identified in multiple studies in our review as increased in CRC patients, while its amino acid precursor, tryptophan, was found to be downregulated across several studies (Table 3). Also often perturbed in CRC are lysophosphatidylcholines (LysoPC), three of which were found downregulated in CRC across multiple studies (Table 3). LysoPC is a class of phospholipid often significantly lowered in CRC cases, and increased breakdown of these groups to phosphatidylcholine groups increases cancer malignancy [75,76]. Phosphatidylcholines, when incorporated into cell membranes, can alter membrane potential and motility, increasing ability of cell adhesion and leading to enhanced malignancy [76]. The mechanism whereby LysoPCs are associated with lowered cancer risk, conversely, likely involves activation of apoptosis-inducing factors, such as caspases and cytochrome c release [77]. Additionally, 3-hydroxybutarate, a ketone body identified to be more prevalent in CRC in 3 of our identified studies, is sometimes also implicated in CRC pathogenesis, where one study found it can the expression of genes responsible for mitochondrial biogenesis, self-renewal, and migration [66].

Additionally, certain mutations leading to CRC may cause differential metabolic profiles due to their different metabolism-related downstream targets, although further clarification through integrated metabolomics and proteomics/transcriptomics are likely needed for full elucidation of these pathways. There have been some preliminary studies demonstrating that APC and KRAS mutations’ characteristic activation of WNT pathways may give rise to a glycolytic phenotype that differs largely from that of mismatch repair mutations [5]. However, metabolic characteristics of CRC stemming from differing genetic mutations, especially in the case of mismatch repair, have not been well-characterized. Nonetheless, this is an area of CRC pathogenesis worthy of further investigation.

### 3.8. Biospecimen-Specific Metabolite Biomarkers in CRC

Some, but not all, dysregulated metabolites were able to be differentiated by specimen types in our collection of studies. Several metabolites were more often identified in stool samples including palmitic acid, lysine, and sphinganine (Table 3). The upregulation of sphinganine and palmitic acid can possibly be tied to the same metabolic pathway, in which palmitic acid, either from dietary sources or endogenous synthesis is converted to sphingolipids either by the host or by some gut microbes in the *Bacteroides* genus by the enzyme serine-palmitoyl transferase [77]. Sphingolipids such as sphinganine have modulatory effects on cancer cells, including increasing proliferation through mediation of sphingosine-1-phosphate [78,79]. Thus, elevated levels of both molecules could increase CRC pathogenesis. Palmitic acid has also been found to independently increase cancer proliferation in some studies by the induction of β-adrenergic receptor expression [62]. Kynurenine was also largely identified in urine samples (Table 3), where it is typically excreted and used in diagnostic tests for cardiovascular disease as a marker of inflammation [80]. Certain lipid derivatives, such as LysoPC’s and 3-hydroxybutyrate (3-HB) were found exclusively in blood-related specimen types (plasma and serum), as these molecules are typically free in circulation when not being taken up by tissues (3-HB) or being incorporated into membranes (LysoPC’s) [81]. Although the data we collected did not demonstrate strong differentiation in every metabolite between different specimen types, metabolic profiles are known to differ even inter-individually between specimen types, which can even somewhat differ between similar specimen types such as serum and plasma [82]. Additionally, concentrations of metabolites in specimens such as urine can vary based on circadian rhythm and diet, and thus standardization of collection time and control for differential patterns of nutrient consumption, as well as specimen type, could be an important consideration in future studies [83,84].

### 3.9. Stool Studies and Gut Microbiota/Microbial Metabolites in CRC

An emerging trend in CRC metabolic biomarker research is to analyze the interplay between these metabolites and the subject’s intestinal microbial composition in stool specimens through genomic analysis methods. This is due to significant correlation between gut microbial composition and CRC incidence, where it is well-known that these bacteria can produce their own oncogenic metabolites and inflammatory factors that may contribute to the overall pathogenesis of CRC [85,86,87]. Several metabolite classes have been previously characterized as up or downregulated in CRC in fecal metabolomic studies, including secondary bile acids, short chain fatty acids (SCFAs), and polyamines [20,88]. Secondary bile acids are produced by bacteria in the gut through modification of endogenously produced primary bile acids [89]. Many secondary bile acids can be carcinogenic and if produced in too high quantities, may lead to pathogenesis of CRC [88,90]. Polyamines, a class of biomolecule containing multiple amino groups typically produced by gut microbes, are often found to be upregulated in cancer pathology due to carcinomas’ increased need for cell growth and proliferation [91]. Conversely, SCFAs (acetate, propionate, and butyrate), typically demonstrate an inverse correlation with CRC incidence, likely acting as anti-inflammatory and apoptosis-inducing factors [88]. Although microbial metabolites typically intermix with all major human biospecimens, the fecal metabolome serves as an especially powerful model of the gut metabolome as many gut metabolites are excreted with stool [92]. Genetic analyses of CRC patients have identified certain bacterial genera as positively associated with CRC, including *Fusobacterium*, *Bacteroides*, and *Enterococcus* [19,93,94]. Negatively correlated genera include *Lachnospiracaea* and *Clostridium* [93,94]. Some of these bacteria were identified in our six identified metabolomic studies using stool samples as the primary biological specimen. For instance, *Fusobacterium* was identified in 16S ribosomal RNA gene sequency to be more abundant in CRC samples by Yang et al. and Sinha et al. while *Clostridium* was found to be less abundant in CRC samples by Yang et al. and Sinha et al. *Lachnospiracaea* was found to be less abundant in CRC samples by Yang et al. and Sinha et al. while found to be more abundant in CRC by Kim et al. (Figure 5a) [33,46,48]. We identified six studies that employed metabolomics to recognize many metabolites associated with CRC incidence and correlated them with corresponding 16S ribosomal RNA gene analyses of gut bacteria genera [33,34,37,44,46,48]. For instance, Kim et al. identified upregulated bile acid-related metabolites in CRC patients including deoxycholate and bilirubin [33]. Weir et al. identified propionic acid (a derivative of SCFA propionate) as differentially regulated in CRC patients, but curiously identified it being upregulated in CRC [44]. Conversely, Yang et al. found propionic acid downregulated in CRC [46]. Additionally, Yachida et al. identified several polyamines as differentially expressed in CRC vs. control patients, including N1,N8-Diacetylspermidine and N1, N12-Diacetylspermine [37]. The most identified (upregulated in CRC) metabolites in stool studies were lysine, sphinganine, and palmitic acid (Table 3), while the most upregulated genera were *Akkermansia* and *Clostridium* (Figure 5a). The metagenomic analyses in conjunction with metabolite measurements were able to generate impressive predictive models of CRC, including Kim et al.’s combined genomic/metabolic dataset generating a diagnostic model exhibiting an AUC of 1.0 [33]. In Figure 5b, we characterize all reported correlations between genomic measurements of bacterial genera identified across five stool specimen studies (Yachida et al. did not report correlation between individual metabolites and bacterial genera), as well as differentially regulated bacterial genera identified in multiple studies [37]. 

### 3.10. Factors That May Influence Metabolic Biomarkers

Metabolomics, contrary to proteomics, genomics, or transcriptomics, represents a transient phenotypic state that may fluctuate rapidly. Metabolites can vary widely by age, weight, sex, diet, and even circadian rhythm [95,96]. This provides both advantages, in that it can reflect a more accurate up-to-the-minute phenotype of the organism, and disadvantages in its sometimes-inconsistent results that can be affected by confounding factors outside those being studied [97]. For instance, CRC incidence as well as gut microbiota makeup can be significantly increased by poor diet and lack of exercise [98,99,100]. These same poor lifestyle factors can lead to a host of other metabolic dysregulation, which may cause CRC-associated metabolic pathway alterations that exist in some patients but not others. Additionally, analytical platform, specimen type, and workflow deviances may affect metabolomics results. Other limitations of this review include lack of a universal, standardized metabolomics workflow for clinical application, regional clustering of studies in Asian populations that may not lead to universal applicability, and lack of external biomarker validation in many studies [101]. In addition to limitations posed by the selected studies, the summary of this systematic review may be limited by imperfect identification of relevant studies, publication bias, or lack of heterogeneity in data reporting or lack of data availability of the individual publications.

### 3.11. Future Directions

Metabolomics represents a promising technique for biomarker identification in CRC pathogenesis. However, its lack of standardization in procedure, biospecimen choice, and use of external validation sets leaves work to be done before defining a standard clinical biomarker panel for diagnosis. We believe this review may serve as a foundation for future studies to conduct more targeted analyses of already identified metabolites in particular biospecimen categories. For instance, our review identified palmitic acid, lysine, and sphinganine to be consistently upregulated in stool, while kynurenine was found to be consistently upregulated in urine (Table 3). The framework we lay out here may guide the eventual progression of metabolomics studies beyond validation stages of biomarker discovery and toward development of clinical metabolomics assays. Additionally, standardization of sample collection and analysis protocols as well as normalization of potentially confounding variables such as BMI, diet, and exercise may assist to drive further homogeneity in biomarker identification. For instance, Zhu et al. included smoking and alcohol consumption of patients as factors driving their predictive model [47].

In addition to diagnosis, metabolomics may be used to drive advances in cancer treatment. For instance, a major hallmark of KRAS mutation-driven CRC is its upregulation of glutaminase 1, which converts glutamine to glutamate [5]. This metabolic dysregulation is largely represented by the data we have collected, showing glutamic acid/glutamate upregulated in CRC in the majority of studies, while glutamine is downregulated in CRC in the majority of studies. Glutaminase 1 inhibitor treatment in many studies has been found to induce cell death and limit proliferation. Administered vitamin C may also have bearing on KRAS driven CRC due to its antioxidant properties, depleting glutathione and leading to arrest of glycolysis [5]. Additional metabolically linked inhibitors of CRC proliferation may be identified by metabolomics with further study. Of additional concern is the elucidation of how metabolites may relate to genomic or protein-related markers of CRC. Some studies have begun to be published in this field, such as a study linking mutated KRAS in mice to increased glutaminolysis and glutamine transport, leading to increased DNA methylation, WNT upregulation, and resistance to 5-fluorouracil [102]. However, this field is still in early stages of development and is certainly in need of additional consideration, especially in the realm of integrated metabolomics, proteomics, and transcriptomics research. 

## 4. Conclusions

Altogether, metabolomics presents a new and innovative method of non-invasive screening for colorectal cancer. As current diagnostic methods are either invasive or lack sensitivity, the utilization of metabolic biomarkers for detection of both colorectal adenomas and adenocarcinomas seems promising. However, current studies have yet to concur on a standard biomarker panel of metabolites. This likely reflects the lack of progression beyond the exploratory or validation stages of metabolite biomarker discovery into later stages of biomarker assay development, such as longitudinal repository or cancer control trials. We propose that a movement into these later stages of biomarker establishment, or at least a use of targeted metabolomics assays for metabolites we have identified as often perturbed in CRC pathogenesis, may soon be warranted, with the caveat that certain metabolite biomarkers may selectively apply to specific biospecimens or conditions. With more consistent workflow for sample collection and instrument usage as well as consistent validation of models, future studies may begin to solidify the differences in the metabolic profiles between colorectal adenomas, adenocarcinomas, and healthy patients, as well as early onset versus late onset colorectal cancer, allowing for progression towards clinical validation of metabolic biomarkers. Additionally, the further characterization of the gut microbiome and metabolome may shed light on metabolic drivers of cancer pathogenesis. Altogether, metabolomics represents a promising method of biomarker detection for colorectal cancer and may present itself as a useful diagnostic tool in the clinical setting in the near future.

## Figures and Tables

**Table 1:** Overview of the 37 articles included in this review. All studies except Geijson et al. and Liu et al. [22,23] compared CRC specimens to either adenoma or normal (healthy) specimens.

Study	Stage	Specimen	Cases & Controls	Platform	Country	Age	% Male	Year	Study Number
Cross et al.	N/A	Serum	CRC: 254, Control: 254	UHPLC-MS, GCMS	America	Median: 64.3	56	2014	[24]
Crotti et. al	Stage I: 11, Stage II: 9, Stage III:16, Stage IV:12	Plasma	CRC: 48, Control: 20	GC-TOF	Italy	49–90 CRC, 35–83 Control	54	2016	[25]
Deng et al.	Stage I: 30, Stage II: 50, Stage III: 57, Stage IV: 31	Urine	Training: CRC: 121, Control: 121; Validation: CRC: 50, Control: 50	LCMS	Canada	Median Control: 58.9, Median CRC: 66.4	55	2019	[26]
Di Giovanni et al.	N/A	Serum	CRC: 18, CRC (Remission): 17, Matched Control: 19 and 17	GCMS	Belgium	32–90 overall, Mean CRC: 70.3, Mean Control: 63.5	24	2020	[27]
Farshidfar et al.	47 Stage I, 60 Stage II, Stage 3: 71, Stage 4: 142	Serum	Training: CRC: 222, Control: 156; Validation: CRC: 98, Adenoma: 31, Control: 98	GCMS	Canada	Mean: Control: 61.7, Adenoma: 59.5, stage I: 68.6, 6 stage II: 68.6 stage III: 64.9, stage IV: 63.1	61	2016	[28]
Geijsen et al.	Stage I: 168, Stage II: 212, Stage III: 290, Stage IV: 74	Plasma	CRC: 744	LCMS	Germany	>18	65	2020	[22]
Gu et al.	N/A	Serum	Training: CRC: 40, Adenoma: 32, Control: 38; Validation: CRC:8, Adenoma: 8, Control: 8	H-NMR	China	N/A	N/A	2019	[29]
Gumpenberger et al.	Stage I: 30, Stage II: 17, Stage III: 18, Stage IV: 12, Unspecified: 3, Missing: 8	Plasma	CRC: 88, High-risk Adenoma: 200, Low-risk Adenoma: 200	UHPLC-MS	America	Mean CRC: 70 Mean HRA: 65.4, Mean Control: 66.0	67	2021	[30]
Holowatyj et al.	Stage I: 40, Stage II: 56, Stage III: 74, Stage IV: 44	Plasma	CRC: 233, Control: 153	LCMS	America	18–89	59	2020	[31]
Jing et al.	Stage I: 10, Stage II: 21, Stage III: 29, Stage IV: 21	Dried Blood Spot	Training: CRC: 77; Adenoma: 73; Testing: CRC: 8, Adenoma: 8	Direct Infusion MS	China	29–79 Adenoma, 22–92 CRC	58	2017	[32]
Kim et al.	Stage I: 7, Stage II: 12, Stage III: 9, Stage IV: 3	Stool	Training: CRC: 26, Control: 32; Validation: CRC: 6, Control: 8	GCMS	South Korea	49–78 Control, 45–80 CRC	58	2020	[33]
Kim et al.	N/A	Stool	CRC: 36, Control: 102, Advanced Adenoma: 102	UHPLC-MS	America	>50	60	2020	[34]
Liu et al.	Stage I/II: 20, Stage III/IV: 20	Plasma	CRC: 40	UHPLC–MS	China	Mean stage I/II: 58.1, Means stage III/IV: 55.25	68	2019	[23]
Long et al.	N/A	Serum	Training: CRC: 30, Adenoma: 30, Control: 30; Validation: CRC: 50, Adenoma: 50, Control: 50	LCMS	America	Mean CRC: 53.97, Mean Adenoma: 51.87, Mean Control: 55.23	53	2017	[35]
Martín-Blázquez et al.	Stage IV: 65	Serum	CRC: 65, Control: 60	LC-HRMS	Spain	Median CRC: 59.9, Median Control: 56.1	52	2019	[36]
Serafim et al.	Stage I: 4, Stage II: 5, Stage III: 31	Plasma	CRC: 40, Adenoma: 12, Control: 32	MALDI-TOF MS	Brazil	Mean Control: 58, Mean Adenoma: 66, Mean CRC: 64	66	2019	[37]
Shu et al.	N/A	Plasma	CRC: 250, Control: 250	GC-TOFMS, UPLC-QTOFMS	China	40–74	50	2018	[38]
Tan et al.	Stage I: 26, Stage II: 43, Stage III: 26, Stage IV: 6	Serum	Training: CRC: 62, Control: 62; Validation: CRC: 39, Control: 40	GC–TOFMS, UPLC–QTOFMS	China	24–82	42	2013	[39]
Uchiyama et al.	Stage I: 14, Stage II: 14, Stage III: 14, Stage IV: 14	Serum	CRC: 56, Adenoma: 59, Control: 60	CE-TOFMS	Japan	Mean Control: 67.7, Mean Adenoma: 69.9, Mean CRC: 70.4	50	2017	[40]
Udo et al.	Stage I: 52, Stage II: 67, Stage III: 71, Stage IV: 17	Urine	Training: CRC: 105, Adenoma: 8, Control: 11; Validation: CRC: 104, Adenoma: 8, Control: 11	LCMS	Japan	Mean Control: 46.8, Mean Adenoma 63.2, Mean CRC: 68.8	58	2020	[41]
Wang et al.	Stage I/II: 61, Stage III/IV: 59	Urine	Training: CRC: 45, Control: 32; Validation: CRC: 10, Control: 8	1H-NMR	China	27–84 Stage I-II, 38–81 Stage III-IV, 28–78 Control	56	2017	[42]
Wang et al.	Stage I: 13, Stage II: 29, Stage III: 27, Stage IV: 4	Plasma	Training: CRC: 34, Control: 34; Validation: CRC: 39, Control: 39	LCMS	China	Mean CRC: 59.7, Mean Control: 57.2	67	2019	[43]
Weir et al.	Stage I:2, Stage II: 3,Stage III: 4	Stool	CRC: 10, Control: 11	GCMS	America	24–85	52	2013	[44]
Wu et al.	N/A	Serum	Colon Cancer: 22, Rectal Cancer: 23, Control: 45	GCMS	China	49–84	69	2020	[45]
Yachida et al.	Stage I/II: 80, Stage III/IV: 68	Stool	CRC: 178, Adenoma: 45, Control: 149, Surgery:34	CE-TOFMS	Japan	Mean Control: 64.11, Mean CRC: 62.04	59	2019	[37]
Yang et al.	Stage I: 9, Stage II: 13, Stage III: 16, Stage IV: 10	Stool	CRC: 50, Control: 50	GCMS	China	N/A	41	2019	[46]
Zhu et al.	Stage I/II: 21, Stage III: 17, Stage IV: 28	Serum	Training: CRC: 46, Adenoma: 53, Control: 64; Validation: CRC: 20, Adenoma: 23, Control: 28	LCMS	America	18–88	48	2014	[47]
Sinha et al.	N/A	Stool	CRC: 42, Control: 89	HPLC-GC/MS-MS	Singapore	Mean: 60 overall	62	2016	[48]
Brown et al.	Stage I: 3, Stage II: 3, Stage III: 8, Stage IV: 1	Stool	CRC: 17, Control: 17	GC-MS, UPLC-MS	America	Mean: 58.8 overall	76	2016	[49]
Goedert et al.	N/A	Stool	CRC: 48, Control: 102	HPLC-GC/MS-MS	America	Mean: 62.9 CRC, 58.3 Control	58.7	2014	[50]
Nishiumi et al.	Stage I: 12, Stage II: 12, Stage III: 12, Stage IV: 12	Serum	Training: CRC: 60, Control: 60; Validation: CRC: 59, Control: 63	GCMS	Japan	36–88, Mean: 67.7	65	2018	[51]
Rachieriu et al.	Stage I: 2, Stage II: 13, Stage 3: 1, Stage IV: 9	Serum	CRC: 25, Control: 16	UPLC-QTOF-ESI+MS	Romania	Mean: 65.9 CRC, 54.2 Control	61	2021	[52]
Lin et al.	Stage I/II: 20, Stage III: 25, Stage IV: 23	Stool	Training: CRC: 54, Control: 26; Validation: CRC: 14, Control: 6	1H-NMR	China	N/A	51	2016	[53]
Ning et al.	Stage II: 65, Stage III: 74, Stage IV: 24	Urine	Training: CRC: 79, Control: 77; Validation: CRC: 76, Control: 30	GC-TOFMS	China	46% under 60, 64% over 60	62.7	2021	[54]
Gao et al.	N/A	Tumor Tissue	CRC: 22, Adenoma: 10	CE-TOFMS	China	N/A	N/A	2016	[55]
Cottet et al.	Stage I: 65, Stage II: 69, Stage III: 55, Stage IV: 14	Adipose Tissue	CRC: 203, Control: 223	GCMS	France	Mean: CRC: 69.5, Control: 66.8	59.7	2014	[56]
Song et al.	Stage I: 3, Stage II: 6, Stage III: 14, Stage IV: 3	Stool	CRC: 26, Adenoma: 27, Control: 28	GCMS	South Korea	Mean: CRC: 59.7, Adenoma: 53.6, Control: 51.1	77.8	2018	[57]

N/A: Not applicable.

**Table 2:** Identified CRC metabolite biomarkers in summarized studies based on their compound class. Metabolite selections were based on the statistical criteria each individual paper set for significance, which listed in the last column. Healthy control vs. CRC data was preferred in studies containing data for both healthy and adenomatous polyp controls.

Name	Study	Specimen	Metabolite Class									Total	Statistical Threshold
			Nucleo-tides	Sterols/Derivates	AA	PA	FA	SL	PL	CHO/Derivatives	Other/Unknowns		
Cross et al.	[24]	Serum			1							1	Bonferroni-corrected *p* value < 0.05
Crotti et al.	[25]	Plasma			4							4	*p* value < 0.05
Deng et al.	[26]	Urine			1	1						2	*p* value < 0.05
Di Giovanni et al.	[27]	Serum			1						2	3	*p* value < 0.1
Farshidfar et al.	[28]	Serum			10		6				8	24	Bonferroni-corrected *p* value < 0.05
Geijsen et al. **	[22]	Plasma			2			2	5			9	FDR-adjusted *p* value < 0.05
Gu et al.	[29]	Serum			13		2			2	6	23	VIP > 1
Gumpenberger et al.	[30]	Plasma	4	2	21		1		13		5	46	FDR-adjusted *p*-value < 0.05
Holowatyj et al.	[31]	Plasma			28			9	75		4	116	FDR-adjusted *p*-value < 0.05
Jing et al. *	[32]	Dried Blood Spot			7		11				3	21	*p* value < 0.05
Kim et al.	[33]	Stool	1	2		1	8	5	3		3	23	FDR-adjusted *p* value < 0.05
Kim et al.	[34]	Stool			5		4				5	14	FDR-adjusted *p* value < 0.1
Liu et al. **	[23]	Plasma		1			7					8	FDR-adjusted *p* value < 0.05
Long et al.	[35]	Serum	2							1		3	*p* value < 0.05
Martín-Blázquez et al.	[36]	Serum						2	2		1	5	FDR-adjusted *p* value < 0.05, VIP > 1
Serafim et al.	[37]	Plasma					4		1		3	8	VIP > 1
Shu et al.	[38]	Plasma	1		2		2		3		1	9	FDR-adjusted *p* value < 0.05
Tan et al.	[39]	Serum		4	23		9	1	7	5	17	66	*p* value < 0.05, VIP > 1,
Uchiyama et al.	[40]	Serum			4		1				2	7	*p* value < 0.01
Udo et al.	[41]	Urine	1		5						3	9	FDR-adjusted *p*-value < 0.05
Wang et al.	[42]	Urine			8						7	15	*p* value < 0.05, VIP > 1,
Wang et al.	[43]	Plasma	2	1	3						1	7	FDR-adjusted *p* value < 0.05, VIP > 1
Weir et al.	[44]	Stool		2	10		5			2	1	20	*p* value < 0.05
Wu et al.	[45]	Serum			5					2	2	9	*p* value < 0.05
Yachida et al.	[37]	Stool	3	2	31	4	5			4	8	57	*p* value < 0.005
Yang et al.	[46]	Stool	3	1	5	2	7			9	26	53	*p* value < 0.05
Zhu et al.	[47]	Serum	1	4	13		4				6	28	*p* value < 0.05, VIP > 1
Sinha et al.	[48]	Stool					1	1			2	4	FDR-adjusted *p* value < 0.05
Brown et al.	[49]	Stool	1	1	13	1	1	1		4	2	24	*p* value < 0.05
Goedert et al.	[50]	Stool			19	1		1			17	38	FDR-adjusted *p* value < 0.1
Nishiumi et al.	[51]	Serum			5					1	4	10	*p* value < 0.05
Rachieriu et al.	[52]	Serum		8			2		20		2	32	*p* value < 0.1
Lin et al.	[53]	Stool			6		3			1	3	13	*p* value < 0.5, VIP > 1
Ning et al.	[54]	Urine			5		2			1	7	15	*p* value < 0.5, VIP > 1
Gao et al.	[55]	Tumor Tissue			9							9	*p* value < 0.001
Cottet et al.	[56]	Adipose Tissue					4					4	*p* value < 0.05
Song et al. ***	[57]	Stool					2					2	*p* value < 0.05

* Study compared CRC to adenomatous polyp rather than healthy control. ** Study compared CRC to other CRC patients (stage specific comparison). *** Only significant in male patients. Abbreviations: AA: Amino Acid, PA: Polyamine, FA: Fatty Acid, SL: Sphingolipid, PL: Phospholipid, CHO: Carbohydrate, VIP: Variable Importance in Projection, FDR: False Discovery Rate

**Table 3:** CRC related metabolite biomarkers identified in 3 or more studies. Summarized metabolite was found increased or decreased in CRC vs. control in each paper, respectively. Reference numbers of studies in which a particular metabolite was identified are contained in parentheses after number of studies demonstrating increase or decrease. Consensus direction is reported in the final column, depicting the most commonly identified direction of regulation of each particular metabolite across reviewed studies.

Metabolite	Times Identified	Studies Showing Upregulation	Studies Showing Downregulation	Studies Not Reporting Regulation Directionality	Consensus Direction
3-hydroxybutarate	4	3 (48, 35, 55)	1 (52)		↑
Alanine	10	4 (53, 54, 22, 42)	6 (48, 36, 46, 35, 28, 52)		↓
Asparagine	6	1 (24)	5 (36, 46, 35, 28, 44)		↓
Aspartic Acid	5	1 (40)	4 (48, 35, 55, 38)		↓
Choline	3	1 (48)	2 (49, 28)		↓
Citrulline	5	2 (51, 56)	3 (23, 36, 45)		↓
Creatinine	3	1 (35)	2 (28, 33)		↓
Cystine	5	3 (35, 51, 40)	1 (53)	1 (56)	↑
Deoxycholate	3	1 (31)	2 (38, 44)		↓
Glucose	4	3 (48, 27, 43)	1 (38)		↑
Glutamic Acid/Glutamate	8	7 (24, 48, 36, 22, 38, 40, 41)	1 (35)		↑
Glutamine	4	1 (28)	3 (48, 36, 42)		↓
Glycerol	5	4 (53, 48, 22, 39)	1 (35)		↑
Glycine	7	6 (53, 48, 22, 28, 56)	1 (43)		↑
Hippurate/Hippuric Acid	3	2 (49, 52)	1 (28)		↑
Histidine	8	8 (23, 36, 35, 55, 33, 52, 44, 43)			↑
Isoleucine	6	6 (53, 48, 54, 27, 56, 47)			↑
Kynurenine	5	4 (30, 51, 56, 40)	1 (36)		↑
Lactate	3	2 (48, 41)		1 (56)	↑
Leucine	5	4 (48, 54, 22, 56)	1 (36)		↑
Linoleic Acid	5	3 (53, 35, 25)	2 (22, 45)		↑
Lysine	6	4 (53, 48, 54, 22)	2 (36, 55)		↑
LysoPC 16:0	3		3 (23, 49, 36)		↓
LysoPC 16:1	3		3 (49, 36, 35)		↓
LysoPC 17:0	3		3 (24, 49, 36)		↓
Methionine	4	1 (47)	3 (36, 35, 52)		↓
Palmitic Acid	5	5 (53, 31, 54, 35, 43)			↑
Phenylalanine	7	3 (53, 56, 42)	4 (36, 35, 33, 56)		↓
Proline	6	4 (49, 38, 42, 47)	2 (48, 36)		↑
Serine	4	2 (48, 22)	1 (35)	1 (56)	↑
Sphinganine	3	3 (31, 32, 35)			↑
Succinate	3	2 (54, 56)	1 (48)		↑
Tryptophan	5	1 (43)	4 (36, 55, 28, 27)		↓
Tyrosine	9	3 (53, 43, 47)	6 (48, 36, 46, 28, 56, 38)		↓
Urea	3	1 (38)	2 (35, 27)		↓
Valine	7	3 (22, 56, 47)	4 (48, 49, 36, 46)		↓

**Table 4:** Metabolic pathways significantly (*p* < 0.05) upregulated or downregulated in CRC across multiple studies, as depicted in Figure 4, along with metabolites significantly up or downregulated in each pathway. Analysis was performed by using MetaboAnalyst version 5.0 (https://www.metaboanalyst.ca/home.xhtml), developed by the Xia lab, Alberta, Canada, accessed on 8 August 2021.

Pathway	Metabolites Implicated	*p* Value	Regulation
Aminoacyl-tRNA biosynthesis	7	5.07 × 10^−8^	Upregulated
Valine, leucine, and isoleucine biosynthesis	2	0.00205	Upregulated
Butanoate Metabolism	2	0.00743	Upregulated
Histidine Metabolism	2	0.00845	Upregulated
Glycolysis or Gluconeogenesis	2	0.0217	Upregulated
Alanine, Aspartate, and glutamine metabolism	2	0.025	Upregulated
Glutathione Metabolism	2	0.025	Upregulated
Porphyrin metabolism	2	0.0285	Upregulated
Glyoxylate and dicarboxylate metabolism	2	0.0322	Upregulated
Biosynthesis of unsaturated fatty acids	2	0.04	Upregulated
Arginine and proline metabolism	2	0.0442	Upregulated
Linoleic acid metabolism	1	0.0444	Upregulated
Valine, leucine, and isoleucine degradation	2	0.0485	Upregulated
Aminoacyl-tRNA biosynthesis	9	2.46 × 10^−12^1	Downregulated
Arginine biosynthesis	4	0.00000198	Downregulated
Alanine, aspartate, and glutamate metabolism	4	0.0000382	Downregulated
Phenylalanine, tyrosine, and tryptophan biosynthesis	2	0.000327	Downregulated
Phenylalanine metabolism	2	0.00239	Downregulated
Pantothenate and CoA biosynthesis	2	0.00873	Downregulated
D-Glutamine and D-glutamate metabolism	1	0.0456	Downregulated
Nitrogen metabolism	1	0.0456	Downregulated
